# Change of Optical Intensity during Healing Process of Corneal Wound on Anterior Segment Optical Coherence Tomography

**DOI:** 10.1038/srep32352

**Published:** 2016-08-26

**Authors:** Kangkeng Zheng, Haifan Huang, Kun Peng, Jianhao Cai, Vishal Jhanji, Haoyu Chen

**Affiliations:** 1 Joint Shantou International Eye Center, Shantou University & the Chinese University of Hong Kong, Shantou, China; 2 Department of Ophthalmology and Visual Sciences, the Chinese University of Hong Kong, Hong Kong, China

## Abstract

The purpose of this study is to investigate the process of corneal wound healing after penetrating injury with the change in optical intensity on anterior segment optical coherence tomography (AS-OCT) and to investigate factors associated with severity of corneal scar. Forty-seven eyes from 47 patients with repaired corneal laceration were included. AS-OCT was performed on 1day, 1week, 1, 3 and 6 months after primary repair. Internal aberrations of wound edges were observed on AS-OCT images. Parameters including height of steps, width of gaps, maximal corneal thickness, area and optical intensity of corneal wound/scar were measured. The relationship between the parameters at day 1 and the optical intensity at 6 months were analyzed. The results showed that optical intensity of corneal wound/scar increased from 124.1 ± 18.8 on day 1 postoperatively to 129.3 ± 18.7, 134.2 ± 23.4, 139.7 ± 26.5, 148.2 ± 26.4 at 1 week, 1 month, 3 months and 6 months postoperatively. Height of steps at 1 day after surgery was the only factor identified as correlated with optical intensity of corneal scar at 6 months (beta = 0.34, p = 0.024). The increase of optical intensity represents the process of fibrosis of corneal wound healing. Higher step after suturing is associated with more severity of corneal scar at last.

Ocular trauma is a leading cause of unilateral blindness^1^. Corneal injuries can cause significant visual impairment^2^. Although corneal wound can be successfully closed with suturing, the resultant corneal scar affects the visual acuity permanently unless corneal transplantation.

Anterior segment optical coherence tomography (AS-OCT) provides cross-sectional images of ocular tissues^3^,^4^. Compared to other imaging modalities such as ultrasonic biomicroscopy (UBM), AS-OCT has the advantages of noncontact nature with high resolution and high repeatability, which makes it a better choice for ocular trauma patients. Utsunomiya^5^ observed the wound healing process after corneal wound thinning in keratitis and corneal foreign bodies with AS-OCT. Opaque scar tissue was detected with slit-lamp examination and was characterized by a high intensity on AS-OCT. Madhusudhana^6^ reported a case of penetrating eye injury and utilized AS-OCT to show the changes in anatomical structures of anterior segment. Wylegala^7^ used AS-OCT to examine 38 eyes with various ocular injuries and suggested it a valuable tool for early diagnosis and monitoring of ocular trauma.

AS-OCT can provide not only high resolution images and measurement of morphological parameters, but also character of tissue, which is represented by the quantitative analysis of optical intensity. This parameter has been used to quantify different retinal layers in retinal diseases^8^ as well as retinal nerve fiber layer (RNFL) in glaucoma^9^. It is also valuable to detect intraretinal and subretinal spaces in choroidal neovascularization^10^,^11^. However, to the best of our knowledge, the usefulness of AS-OCT to quantify corneal wound with optical intensity has not been reported on literature.

In the current study, we used AS-OCT to investigate corneal wound healing with the change in optical intensity. We also aimed to identify factors associated with the severity of corneal scar.

## Results

A total of 47 patients with full-thickness corneal laceration were included. The mean age was 33 ± 12 years (range 6–51 years). Most (89%) of the patients were males.

**Figure 1:**
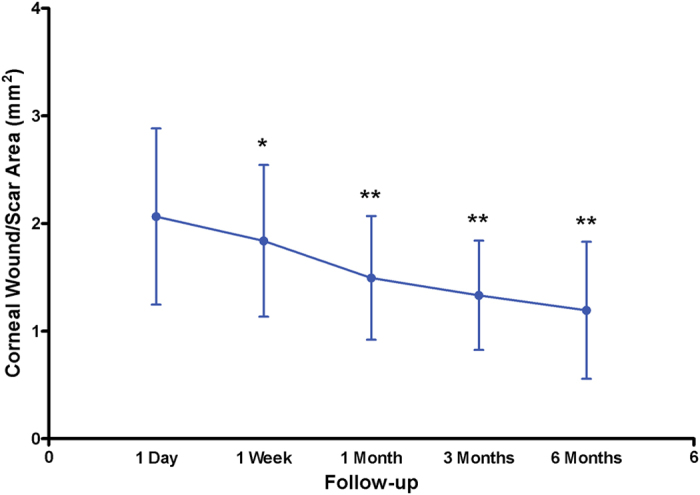
Change of corneal scar area on anterior segment optical coherence tomography with time after surgery. Asterisks signify statistically significant difference compared to corneal wound area at 1 day using paired-samples t test.(*p < 0.05, **p < 0.001).

**Figure 2:**
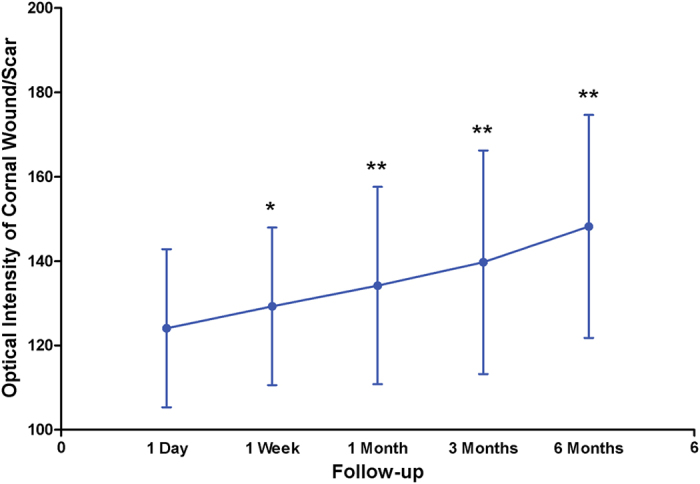
Change of optical intensity of corneal scar on anterior segment optical coherence tomography with time after surgery. Asterisks signify statistically significant difference compared to optical intensity of corneal wound at 1 day using paired-samples t test.(*p < 0.05, **p < 0.001).

On AS-OCT, the corneal wound/scar area was 2.064 ± 0.819 mm^2^, 1.839 ± 0.705 mm^2^, 1.495 ± 0.575 mm^2^, 1.332 ± 0.507 mm^2^ and 1.193 ± 0.637 mm^2^ at 1 day, 1 week, 1 month, 3 months and 6 months after suturing respectively (Fig. 1). The optical intensity of wound/scar was 124.1 ± 18.8, 129.3 ± 18.7, 134.2 ± 23.4, 139.7 ± 26.5 and 148.2 ± 26.4 at 1 day, 1 week, 1 month, 3 months and 6 months after suturing respectively (Fig. 2). The reduction of wound area and the increase of optical intensity among all follow-up times are statistically significant.

**Figure 3:**
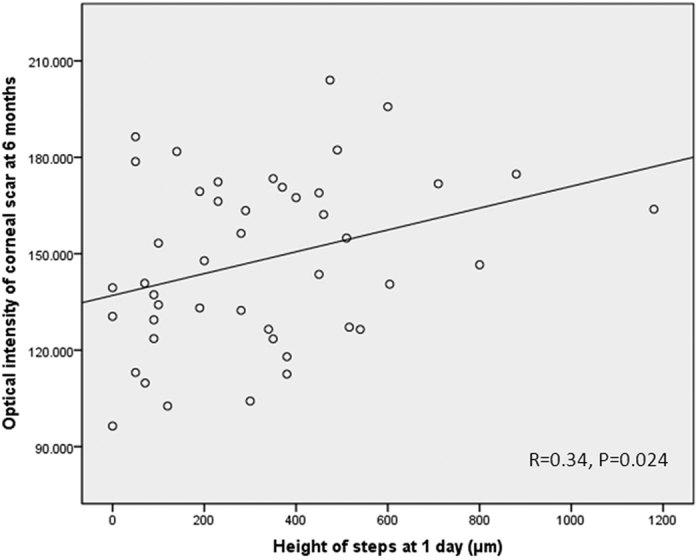
Scatter plots of height of steps at 1 day against optical intensity of wound/scar at 6 months.

**Figure 4:**
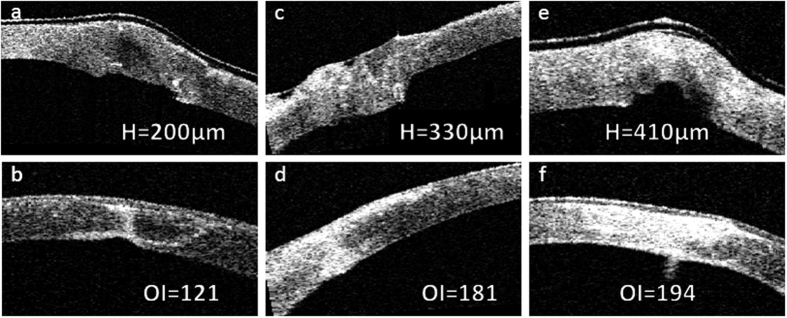
Representative cases showing different height of step at one day after surgery (**a,c,e**) and their outcome with different severity of corneal scar at 6 months (**b,d,f**) after surgery. H: height of step, OI: optical intensity.

Univariate analysis shows that height of steps at postoperative day 1 was associated with the final optical intensity of scar (r = 0.34, p = 0.024, Fig. 3). There was no statistically significant association between gender, age, corneal wound area, width of gaps or maximal corneal thickness with the final optical intensity of corneal scar. Table 1 lists relationship between optical intensity of corneal scar at 6 months with various parameters at 1 day in univariate analysis. In multivariate linear regression, height of steps was the only factor correlated with the optical intensity of scar at 6 months (beta = 0.34, p = 0.024). Figure 4 illustrates 3 examples of corneal wounds on AS-OCT. As the height of steps increased, the value of optical intensity of corneal scar increased.

## Discussion

In this study, AS-OCT demonstrates decrease in wound area and increase in optical intensity during healing process after suturing of traumatic corneal injury. Furthermore, we identified that height of step at 1 day postoperatively was positively correlated with optical intensity of the scar at 6 months.

Previously, AS-OCT has been used to assess anterior segment wounds and several studies have highlighted its advantages of noncontact nature and penetrating capability to detect corneal injuries^3^,^12^,^13^. This device is also valuable to observe graft-host appositions after penetrating keratoplasty (PKP)^14^,^15^,^16^. Both PKP wounds and traumatic corneal wounds have corneal suture placement and similar wound appositions may occur after suturing. This may be explained by the unsutured endothelium resulting from partial thickness suturing. Uneven corneal edema of the wound edges may be another causative factor. Such appositions can be well detected by AS-OCT, which can facilitate diagnosis and treatment of these corneal wounds.

AS-OCT utilizes 1310 nm wavelength to compare backscatter light of tissue with the reference path^17^. Highly organized collagen fibrils of the cornea reduces light scatter^18^. Thus, transparent cornea is exhibited as middle signal on AS-OCT. Corneal injury activates adjacent keratocytes to and causes their migration to the damaged area, followed by transformation to fibroblasts and myofibroblasts^19^. These two cell types are associated with corneal opacity^19^,^20^. In long term, disorganized fibrotic tissue will deposit and is seen as hyperreflective space on AS-OCT, corresponding to the increased optical intensity. McCally^21^ has reported that disordered, enlarged fibrils contributed to light-scattering properties of corneal scar secondary to penetrating wounds in rabbit cornea. Our quantitative analysis of the scans revealed that optical intensity of wound increased with time, which indicates the process of corneal repairing and fibrosis.

A statistically significant correlation was found between height of steps at 1 day and optical intensity of corneal scar at 6 months. Compared to daily renewal of corneal epithelium, endothelial repair takes much longer time. Three stages are involved, consisting of cell migration, restoration of endothelial pump and cell remodeling, which can continue for months^18^. For wounds with steps, misalignment of wound edges will further slow down the healing process of endothelium and cause more fibrosis of the wound, which is presented as higher optical intensity on AS-OCT. This correlation also suggests the importance of avoiding steps by careful suturing.

Our study was the first to show that optical intensity is a good biomarker that shows changes of corneal wound during the healing process and expands the application of optical intensity in ocular diseases.

We recognize some limitations in our study. The sample size is relatively small. The AS-OCT images were acquired only from 1 cross-section of the wound, so they could not show the 3-dimentional architecture of the lesion. Although we tried to scan the same area on AS-OCT examination at each follow-up, the results would be more reliable if a tracing system is available. In addition, the study subjects had various severity of injury due to the nature of ocular trauma.

In conclusion, our study found that optical intensity of corneal wound increases with time, which may represent the process of fibrosis. We also found the height of steps on AS-OCT imaging at 1 day postoperatively was correlated with optical intensity of corneal scar at 6 months. Despite the observation of wound repair process with optical intensity, further studies are needed to confirm the fibrotic process using anterior segment imaging modalities.

## Methods

This study was adhered to the tenets of Declaration of Helsinki, and approved by the institutional review board of Joint Shantou International Eye Center (JSIEC) of Shantou University and Chinese University of Hong Kong. It was a prospective case series study. Informed consent was obtained from all participants. Forty-seven eyes from 47 patients were enrolled between February 2012 and July 2014. All patients had traumatic full-thickness corneal wound and received corneal suturing surgery in JSIEC. Patients with history of prior ocular surgery, trauma, infection and history of diabetes or connective tissue diseases were excluded. The patients with intraocular tissue adhered to the corneal wound were also excluded because it may affect the healing process of wound.

### Surgical technique

All surgeries were performed in the same hospital using a standard surgical technique. Under topical anesthesia, deep interrupted 10–0 nylon sutures were placed 1.5–2.0 mm apart with identical bite length on both sides of the wound. All knots were buried and wounds were checked for leak at the end of the surgery. One day after surgery, patients were instructed to instill tobramycin and prednisolone eye drops every 2 hours for 1 week and reduced to 4 times daily for 3 weeks. Suture removal was performed 3 months after the surgery.

### Anterior segment OCT analysis

**Figure 5:**
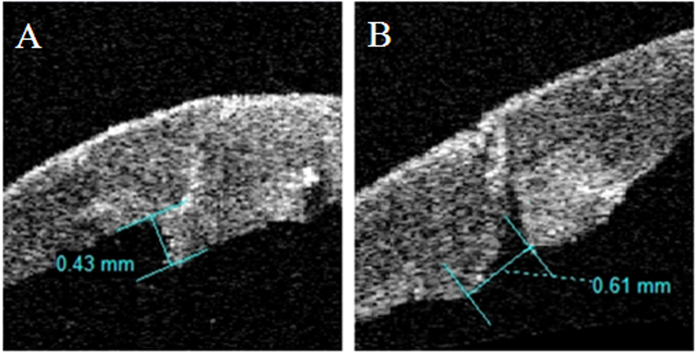
AS-OCT images show the measurement of height of step (**A**) and width of gap (**B**) for corneal wound after suture. (**A**) Height of step was the difference between the two vertical distances from the outer corneal boundary to the inner ends of the wound. (**B**) Width of gap was the distance between the non-apposed inner wound edges.

AS-OCT (Visante OCT; Carl Zeiss Meditec, Inc, Dublin, CA) was performed at 1 day, 1 week, 1 month, 3 months and 6 months postoperatively. All patients were scanned with high-resolution optical sections. Lines crossing the central cornea were used to scan the corneal wounds every 5° apart. The scanning degree was set up as the angle between the line crossing the center of the cornea and the horizontal line. Among the sections taken from the wound, images with highest steps were chosen for measurement of parameters and the degrees of these sections were used for the follow-up visits. Any wound apposition was recorded and the discrepancy between the sutured wound edges was measured. The flap tool of the built-in software was employed for the measurement of maximal corneal thickness, height of steps and width of gaps. By automatically recognizing the anterior boundary of the cornea, the flap tool can set any lines perpendicular to the boundary and calculate the distance between the anterior border and the posterior border of the cornea. Height of steps was defined as distance perpendicular to the anterior corneal surface between the two sutured wound edges. Width of gaps was distance between the unsutured inner wound edges. These parameters were measured on Visante OCT Software Version 2.0.1.88 (Fig. 5) by the same technician.

**Figure 6:**
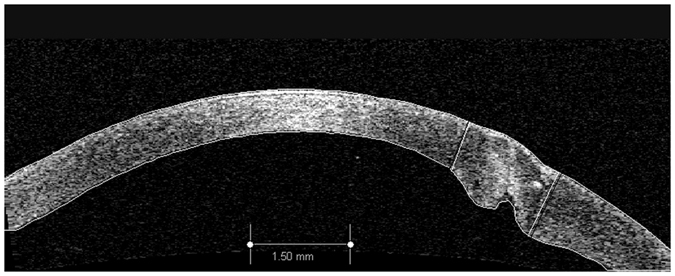
Measurement of optical intensity and area of corneal wound. Two straight lines perpendicular to the anterior corneal surface were drawn to line out the corneal wound/scar, and area and optical intensity of the wound/scar were calculated by the software Image J.

All AS-OCT images were exported and analyzed with Image J (Version 1.48, National Institutes of Health, Bethesda, MD). Figure 6 illustrates the method of corneal wound/scar area and optical intensity measurement. First the boundaries of the anterior and the posterior corneal surfaces were identified. Then, by defining the corneal area as areas with optical intensity changes, two straight lines perpendicular to the anterior corneal surface were used to line out the area of corneal wound/scar. At last, area and optical intensity of the wound scar were automatically calculated by the software. Optical intensity of the wound is the average grey value of the defined area.

### Statistical Analysis

Mean and standard deviation were used to evaluate demographic values as well as various parameters measured on AS-OCT. Paired-samples t test was used to compare corneal wound area and optical intensity of the wound at 1 day with other follow-up times. Pearson correlation and Student’s independent t test were used to investigate the relationship between the optical intensity of corneal scar at 6 months and the morphological parameters of corneal wound at postoperative 1 day and demographic factors. Multivariate stepwise linear regression was employed to identify the independent factor for optical intensity at 6 months. SPSS 21.0 software was used for all analyses. P values < 0.05 were considered statistically significant.

## Additional Information

**How to cite this article**: Zheng, K. *et al*. Change of Optical Intensity during Healing Process of Corneal Wound on Anterior Segment Optical Coherence Tomography. *Sci. Rep.* **6**, 32352; doi: 10.1038/srep32352 (2016).

## Figures and Tables

**Table 1:** Correlation of optical intensity of corneal scar at 6months with various parameters at 1 day postoperatively.

Parameters measured at 1 day	Correlation coefficient*	P
Gender	—	0.595
Age	0.129	0.393
Wound/scar area	0.277	0.065
**Height of steps**	**0.340**	**0.024**
Width of gaps	0.019	0.903
Maximal corneal thickness	0.019	0.901

Values in bold are statistically significant.

^*^Student’s independent t test was used to evaluate the relationship between optical intensity of corneal scar at 6months with gender. Other parameters were evaluated with Pearson correlation.
